# Dataset of policy actors during COVID-19 pandemic handling and economic recovery in Bandung city

**DOI:** 10.1016/j.dib.2025.111841

**Published:** 2025-06-27

**Authors:** Ahmad Zaini Miftah, Ida Widianingsih, Entang Adhy Muhtar, Ridwan Sutriadi

**Affiliations:** a Public Finance Administration Study Program, Applied Science Department, School of Vocational Studies, Universitas Padjadjaran Jatinangor, Sumedang 45363, Indonesia; b Public Administration Department, Faculty of Social and Political Sciences, Universitas Padjadjaran, Jalan Ir. Soekarno Km. 21 Jatinangor, Sumedang 45363; Jalan Bukit Dago Utara No. 25, Bandung 40135, Indonesia; c Center for Decentralization and Participatory Development Research, Faculty of Social and Political Sciences, Universitas Padjadjaran, Jatinangor, Sumedang 45363; Jalan Bukit Dago Utara No. 25, Bandung 40135, Indonesia; d Regional and Urban Planning Department, School of Architecture, Planning, and Policy Developments, Institut Teknologi Bandung, Jalan Ganesha No. 10, Bandung 40132, Indonesia

**Keywords:** Policy integration, Social network analysis, COVID-19, Centrality

## Abstract

Every government confronts a health crisis as it grapples with the trade-off between bolstering the health system and safeguarding the economy to mitigate the transmission of COVID-19. The COVID-19 pandemic, which affects multiple sectors, necessitates a responsive, coordinated, and efficient policy approach that involves multiple actors from both central and local government. Thus, policymakers in the healthcare sector play the most central role in handling the COVID-19 pandemic. This article presents a dataset of actors involved in handling the COVID-19 pandemic in Bandung City, including a list of actor positions, roles, nodes, and edges of the actors. The primary data for this study were collected through direct interviews with individuals considered pertinent to the necessary information. Additionally, secondary data were obtained from desk studies on several supporting documents that can validate map actors’ centrality in handling the COVID-19 pandemic in Bandung City. This dataset can be utilized for studying network dynamics in actor mapping, conducting comparative research, and obtaining insights into patterns of interaction, collaboration, and possible conflict related to cross-sectoral issues.

Specifications Table

**Table:** 

Subject	*Social Sciences*
Specific subject area	*Political Science: It examines the complex network of relationships that establish power hierarchies, lobbying networks, collaborations, interaction, and influence policymaking. This is essential for understanding the governance mechanisms and the multifaceted strategies employed by various stakeholders to influence decision-making and results.*
Data format	Raw, Analysed
Type of data	Table
Data collection	The primary data were collected through direct interviews using snowball sampling with individuals considered pertinent to the required information. Data were obtained from several key informants, such as the head of Data and information of the communication and information office (Diskominfo, Dinas Komunikasi dan Informatika), the head of Research and Development of City Planning, Research and Development Office (Bappelitbang, Badan Perencanaan, Penelitian, dan Pengembangan), and government officials with functional positions as researchers at Bappelitbang. Interviews were also conducted via several teleconferences with informants at the provincial and national levels. In addition, secondary data were collected from an examination of various supporting documents that can assist in mapping the centrality of actors involved in dealing with the COVID-19 pandemic in Bandung city.
Data source location	Bandung City Government Office Complex (Bandung City Hall), Jalan Wastukancana No. 3, Bandung City, West Java Province, Indonesia 40117. Lattitude (DD: -6.911000) (DMS: S6^o^ 54’39.6”) Longitude (DD: 107.609880) (DMS: E107 ^o^ 36’35.568”)
Data accessibility	Repository name: Mendeley Data Data identification number: 10.17632/typf4rgbkm.1 Direct URL to data: https://data.mendeley.com/datasets/typf4rgbkm/2
Related research article	Miftah, A. Z., Widianingsih, I., Muhtar, E. A., & Sutriadi, R. (2024). Behind the scenes of COVID-19 response: a social network analysis of policy actors in Bandung City. Cogent Social Sciences, 10(1). 10.1080/23311886.2024.2356351

## 1 Value of the Data

• This dataset contains vast information on diverse policy actors from both the public and private sectors, as well as their relationships and interactions throughout the COVID-19 pandemic.
• It offers data sources that help comprehend the dynamics and efficacy of policy actions taken by the local government during health crises due to the COVID-19 pandemic.
• This dataset could be reused in several ways, including but not limited to comparative studies, longitudinal studies, policy analysis, and network analysis.
• The dataset was provided in an edges-and-nodes format, to ensure accessibility and ease of transformation into application such as the Gephi Software, allowing researchers to analyze and re-visualize it.

## 2 Background

The dataset was initially obtained and employed for a Social Network Analysis (SNA) [1,2] study on policy actors at the local government involved in addressing the COVID-19 pandemic and economic recovery at Bandung City, West Java, Indonesia [3,4]. The data was utilized to analyse the actor centrality in the network structure for managing the COVID-19 pandemic. This involved automated calculations of centrality values and modularity classes, which were then used to build maps using network mapping software recognized as Gephi version 0.101 [5].

Mapping out the involved actors is essential to facilitate planning for integration and collaboration [6]. Mapping is also quite helpful in clarifying the authority of each policy actor and the interaction pattern carried out during the health crisis due to the COVID-19 pandemic. Moreover, this dataset can serve as a foundation for further research and analysis, enabling a deeper understanding and the development of innovative approaches in anticipating the complexity of cross-sectoral issues in the future.

## 3 Data Description

The dataset consists of an excel worksheet (.xlsx), which contains a list of actors, actor codes, and actor roles (Table 1). Comma Separated Values (.csv) files, which contain edges and nodes (Table 2), and ZIP archives (.zip), which contain compressed combined files from the dataset (Table 3). Files containing lists, codes and actor roles were obtained from interviews validated through documents such as Decree of the Mayor of Bandung city No. 440 of 2020 concerning the establishment of a Policy committee, the COVID-19 task Force, and Economic Recovery Task Force in Bandung city, Decree of the Mayor of Bandung city Number 443/Kep.239-Dinkes/2020 concerning COVID-19 Response acceleration task Force in Bandung city as the initial product of the formation of a COVID-19 handling unit, and Presidential Regulation Number 82 of 2020 concerning the COVID-19 Economic Recovery Committee. This data contains three different task forces containing policy actors and networks, namely the task force based on the first policy, Number 443/Kep.239-Dinkes/2020, the task force based on the Decree of the Mayor of Bandung City No. 440 of 2020 as a replacement for the first policy, and the economic recovery task force. Table 1 presents all policy actors involved and their roles. If an actor contributes to more than one task force, the actor is still listed for each task force to reflect the different roles carried out.

**Table 1:** Description of the dataset file (.xlsx)

File Name	Worksheet Name	Description
01_ActrLst	AL Managing COVID-19	The worksheet comprises a list of actors involved in managing the COVID-19 pandemic in Bandung City. It contains the names of government officials, their corresponding codes, and the roles they play.
	AL Economic Recovery	The worksheet includes a list of actors, together with their public positions, codes, and roles in the economic recovery efforts in Bandung City in response to the COVID-19 pandemic.

**Table 2:** Description of the dataset file (.csv)

File Name	Description
02_Ed_GTC-19	This file is a Comma Separated Values (.csv) file type that contains the networks and relationships of actors during Bandung's initial handling of the COVID-19 pandemic (April 2020 - December 2020).
03_Nd_GTC-19	This file is a Comma-Separated Values (.csv) file type that contains actor nodes from the initial period of handling the COVID-19 pandemic in Bandung City (April 2020 - December 2020).
04_Ed_STC-19	This file is a Comma-Separated Values (.csv) file that contains the network and relationships of actors in Bandung City from December 2020 to June 2023, namely during the mid-to-late period until the COVID-19 pandemic restrictions were eased in June 2023.
05_Nd_STC-19	This file is a Comma-Separated Values (.csv) file that contains the nodes of actors in Bandung City from December 2020 to June 2023, namely during the mid-to-late period until the COVID-19 pandemic restrictions were eased in June 2023.
06_Ed_Coll_PVSTC-19	It is a Comma-Separated Values (.csv) file that includes the network and connections between actors from the public and private sectors who are engaged in the economic recovery efforts in Bandung as a response to the COVID-19 pandemic issue.
07_Nd_Coll_PVSTC-19	It is a Comma-Separated Values (.csv) file that includes the nodes between actors from the public and private sectors who are engaged in the economic recovery efforts in Bandung as a response to the COVID-19 pandemic issue.

**Table 3:** Description of the dataset file (.zip)

File Name	Description
11_PAC-19	This file is a compressed file in the format of a .zip file. It contains the complete dataset, including all its contents: 1. 01_ActrLst.xlsx 2. 02_Ed_GTC-19.csv 3. 03_Nd_GTC-19.csv 4. 04_Ed_STC-19.csv 5. 05_Nd_STC-19.csv 6. 06_Ed_Coll_PVSTC-19.csv 7. 07_Nd_Coll_PVSTC-19.csv

## 4 Experimental Design, Materials and Methods

The data for this study were obtained through direct interviews involving individuals considered pertinent to this study placed into the node and edges data in the attached link. Additionally, interviews were carried out through multiple teleconferences with informants at both the provincial and national levels. In addition, in listing all actors involved, this study also refers to relevant regulations, namely the Decree of the Mayor of Bandung City No. 440 of 2020 concerning the Establishment of a Policy Committee, the COVID-19 Task Force, and the Economic Recovery Task Force in Bandung City. Furthermore, supplementary data were gathered by scrutinizing diverse supporting documents that could be beneficial in delineating the prominence of individuals engaged in addressing the COVID-19 pandemic in Bandung city.

The interview process was an open question with the following interview guidelines:

1. What is your role in responding to the COVID-19 pandemic in Bandung city?
2. Who are the key actors involved in policies and actions related to COVID-19 in Bandung and in contact with you?
3. What is the relationship between these actors when making decisions and implementing policies?
4. Are there differences in social networks based on their sector in response to COVID-19?
5. How do collaboration and communication between policy actors influence the effectiveness of the COVID-19 response in Bandung City?

This study applies social network analysis (SNA) [1] To describe the interconnectedness among actors and organizations engaged in managing the COVID-19 pandemic in Bandung city. This approach involves analyzing the structure of social networks that are established among individuals in a social setting, where these individuals are related to each other either directly or undirected [7,8]. Edges with a directed type have an arrow at one end, showing that the relationship is asymmetric, whereas undirected edges are regular lines, indicating a symmetric relationship [1].

The degree of an actor node is the number of network links (edges) that connect it. This analysis is based on assumptions about the relevance of these edges [1]. In this study, the edges were derived from the findings of interviews and related policies regarding the roles and nature of the relationships between the actors. The nodes in these datasets are grouped into three categories: big, medium, and small, based on observations, interviews with informants, and policy document reviews. This categorization is intended to reflect the roles of the actors as a contextual understanding of the nodes within the network. Degree, closeness, and betweenness are the number of relationships a node has with other nodes, how quickly it can reach all its peers, and how often it is on the shortest path between two nodes. Degree, or how many connections a node has, measures centrality in interaction graphs. Centrality calculations in this study utilize degree centrality and closeness. The formula utilized for calculation is as follows.

### 4.1 Degree centrality

 

$$∁\begin{array}{c} \;\\ D \end{array}\left(ni\right)=d\left(ni\right)$$

Description: d(ni) is the number of interactions or relationships that one node has with other nodes in the same network.

### 4.2 Closeness centrality

$$∁\begin{array}{c} \;\\ C \end{array}\left(ni\right)=\left[N-1\;/\;\sum \;d\left(ni,\;nj\right)\right]$$

Description:

*N* = the total number of nodes in a network d*(ni, nj)* = the number of shortest paths connecting nodes *ni* and *nj*

Thus, Gephi version 0.101 was used to automatically construct maps of actor centrality in the network structure after processing the centrality value and modularity class the calculations.

## Limitations

The primary constraint of this dataset is its limited scope, as it solely records the interactions inside the actor network, with a particular focus on actors from the public sector who exerted dominance. When addressing complex issues like the health crisis created by the pandemic, it is crucial to consider the significant influence of the business sector and other stakeholders. To overcome these restrictions, future study should focus on collecting data from a wide range of sources, such as social media platforms and other relevant sources.

## Ethics Statement

The author has adhered to the ethical guidelines for publication in Data in Brief. The research does not encompass any behavioural interventions or manipulations on human subjects. Additionally, the dataset provided does not include any personal information. The Research Ethics Committee of Padjadjaran University has approved this research with Letter Number 1035/UN6.KEP/EC/2024 and Registration Number 2407071039.

## Credit Author Statement

**Ahmad Zaini Miftah:** Conceptualization, Validation, Formal analysis, Investigation, Writing - Original Draft **Ida Widianingsih:** Conceptualization, Methodology, Validation, Formal analysis, Investigation, Writing - Review & Editing, Supervision **Entang Adhy Muhtar:** Validation, Formal analysis, Writing - Review & Editing **Ridwan Sutriadi:** Validation, Formal analysis Writing - Review & Editing.

## Data Availability

Mendeley DataDataset of Policy Actors During COVID-19 Pandemic Handling and Economic Recovery in Bandung City (Original data).
